# Prevalence, risk factors and molecular epidemiology of carbapenem-resistant *Klebsiella pneumoniae* in patients from Zhejiang, China, 2008–2018

**DOI:** 10.1080/22221751.2020.1799721

**Published:** 2020-08-03

**Authors:** Yanyan Hu, Congcong Liu, Zhangqi Shen, Hongwei Zhou, Junmin Cao, Shi Chen, Huoyang Lv, Mingming Zhou, Qiang Wang, Long Sun, Qiaoling Sun, Fupin Hu, Yang Wang, Rong Zhang

**Affiliations:** a Clinical Microbiology Laboratory, 2nd Affiliated Hospital of Zhejiang University, School of Medicine, Zhejiang University, Hangzhou, People’s Republic of China; b Beijing Advanced Innovation Center for Food Nutrition and Human Health, College of Veterinary Medicine, China Agricultural University, Beijing, People’s Republic of China; c Beijing Key Laboratory of Detection Technology for Animal-Derived Food Safety, College of Veterinary Medicine, China Agricultural University, Beijing, People’s Republic of China; d Department of Hospital Infection Control, Zhejiang Provincial Hospital of Traditional Chinese Medicine, Hangzhou, People’s Republic of China; e Clinical Microbiology Laboratory, Hangzhou Third people’s Hospital, Hangzhou, People’s Republic of China; f Clinical Microbiology Laboratory, Zhejiang Provincial People's Hospital, Hangzhou, People’s Republic of China; g National Clinical Research Center for Child Health, The Children's Hospital, Zhejiang University School of Medicine, Hangzhou, People’s Republic of China; h Department of Clinical Laboratory, Hangzhou Maternity and Child Health Care Hospital, Hangzhou, People’s Republic of China; i Institute of Antibiotics, Huashan Hospital, Fudan University, Shanghai, People’s Republic of China

**Keywords:** Carbapenem-resistant, Klebsiella pneumoniae, risk factor, surveillance, molecular epidemiology

## Abstract

Carbapenem-resistant *Klebsiella pneumoniae* (CRKP) is emerging as a worldwide public health concern; however, the long-term molecular epidemiological surveillance of clinical CRKP in China is limited. We conducted a retrospective observational study (2008–2018) to assess the prevalence, susceptibility, risk factors and molecular epidemiology of clinical CRKP isolates. We found the prevalence of CRKP increased from 2.5%, 2008 to 15.8%, 2018. CRKP were significantly more frequent among hospitalized patients from ICU, and it was significantly more likely to be isolated from the capital city (Hangzhou) and the patients aged ≥60 years. Additionally, seasons and specimen types were associated with CRKP infections*.* The main CRKP sequence type (ST) was ST11, and *bla*_KPC-2_ was the most prevalent gene variant. Together these data reveal an increasing incidence and resistance trends among CRKP, especially the ST11-*bla*_KPC-2_-CRKP, in Zhejiang, during 2008–2018. Our findings are important for hospitals to limit its dissemination and optimize antibiotic administration.

## Introduction

*Klebsiella pneumoniae* is one of the most common Gram-negative pathogens associated with clinical infections such as pneumonia, urinary tract infection, sepsis, wound infection, and meningitis [1]. Carbapenems and other β-lactam antibiotics are the commonly used agents to treat *K. pneumoniae* infections, and are also last resort drugs for the treatment of multidrug-resistant bacterial infections. Carbapenem-resistant *K. pneumoniae* (CRKP) was first reported in the 1990s [2,3], and CRKP strains were sporadically isolated throughout that decade [2,3]. However, with the increasing clinical use of carbapenems in recent years, the prevalence of CRKP has risen at an alarming rate, and the pathogen is now considered a serious threat to human health worldwide [4].

In 2016, the World Health Organization was requested to create a priority list of antibiotic-resistant bacteria to support research and development of effective drugs. As expected, CRKP was listed as one of the critical-priority bacteria [5], while carbapenem-resistant Enterobacterales (CRE) were listed as an urgent threat by the United States Centers for Disease Control (https://www.cdc.gov/hai/organisms/cre/). Mortality rates for CRKP infections are higher than those for patients infected with carbapenem-susceptible *K. pneumoniae* (CSKP) [6], with morbidity and mortality rates for CRKP-infected patients in intensive care units (ICU) much higher than those for non-ICU patients [7].

According to the China Antimicrobial Surveillance Network (CHINET), the prevalence of imipenem-resistant *K. pneumoniae* has increased each year in China, from 3.0% in 2005 to 25.0% in 2018, with Zhejiang Province reporting one of the highest rates of resistance (CRKP >50%) in China in 2018 (http://www.chinets.com/). A nationwide report showed that the most common carbapenem resistance mechanism among CRKP strains is the production of carbapenemases. *K. pneumoniae* carbapenemases (KPC) and New Delhi β-lactamases (NDM) are the two main types of enzymes produced by CRKP worldwide, and KPC-2-producing sequence type (ST) 11 *K. pneumoniae* strains are widely disseminated throughout China [8]. Notably, KPC-2-producing *K. pneumoniae* in China was first reported in 2007 in Hangzhou, Zhejiang Province [9], and *bla*_KPC-2_ has since become the most widely spread carbapenemase gene in Zhejiang [10] and China as a whole [11]. Given the clinical importance of CRKP and its high prevalence in Zhejiang Province, an epidemiological analysis of CRKP in Zhejiang in recent years is imperative. Moreover, *K. pneumoniae* is thought to be a key reservoir and transmission vehicle of clinically important antimicrobial resistance genes [12]. Thus, a better understanding and monitoring of these isolates could help limit the spread of antimicrobial resistance and prolong the life of new antibiotics. Herein, we investigated the prevalence and risk factors of CRKP in Zhejiang Province from 2008 to 2018 and an analysis of the main mechanisms of carbapenem resistance were conducted to provide insight into hospital infection control and clinical antimicrobial therapy of CRKP.

## Results

### General information on the surveillance data

Throughout the 11-year surveillance period, although the numbers of participating hospitals and isolates varied, *K. pneumoniae* remained the second-most frequently isolated *Enterobacterales* species each year, accounting for approximately 20.0% of all Gram-negative bacteria. *K. pneumoniae* rank first position in *Enterobacterales* in most hospitals located in Hangzhou or tertiary hospitals were higher than that in non-Hangzhou and secondary hospitals (data not shown). From 2008 to 2018, an average of 30,862 *K. pneumoniae* isolates were collected each year (Table 1). Sputum accounts for the most (approximately 75%) of the clinical samples. The *K. pneumoniae* together with CRKP collected from all the specimen types showed an increasing trend in the past 11 years.

**Table 1.** General information on hospitals and *Klebsiella pneumoniae* isolates.

Year	No. of hospital	No. of isolates	Gram-negative bacteria		*K. pneumoniae*	
			No.	%^a^	No.	%^b^
2008	78	236,301	131,039	55.45	24,734	18.88
2009	95	290,724	173,824	59.79	32,556	18.73
2010	65	230,214	165,202	71.76	30,482	18.45
2011	36	195,030	110,577	56.70	12,707	11.49
2014	48	242,049	129,130	53.35	24,835	19.23
2015	80	302,042	166,141	55.01	31,870	19.18
2016	88	399,629	210,678	52.72	38,547	18.30
2017	80	351,764	208,892	59.38	39,505	18.91
2018	83	323,570	220,140	68.03	42,522	19.31

^a^Percentage of Gram-negative bacteria among total number of reported isolates.

^b^Percentage of *K. pneumoniae* isolates among Gram-negative bacteria.

### Antimicrobial resistance patterns among *K. pneumoniae* isolates

**Figure 1.**
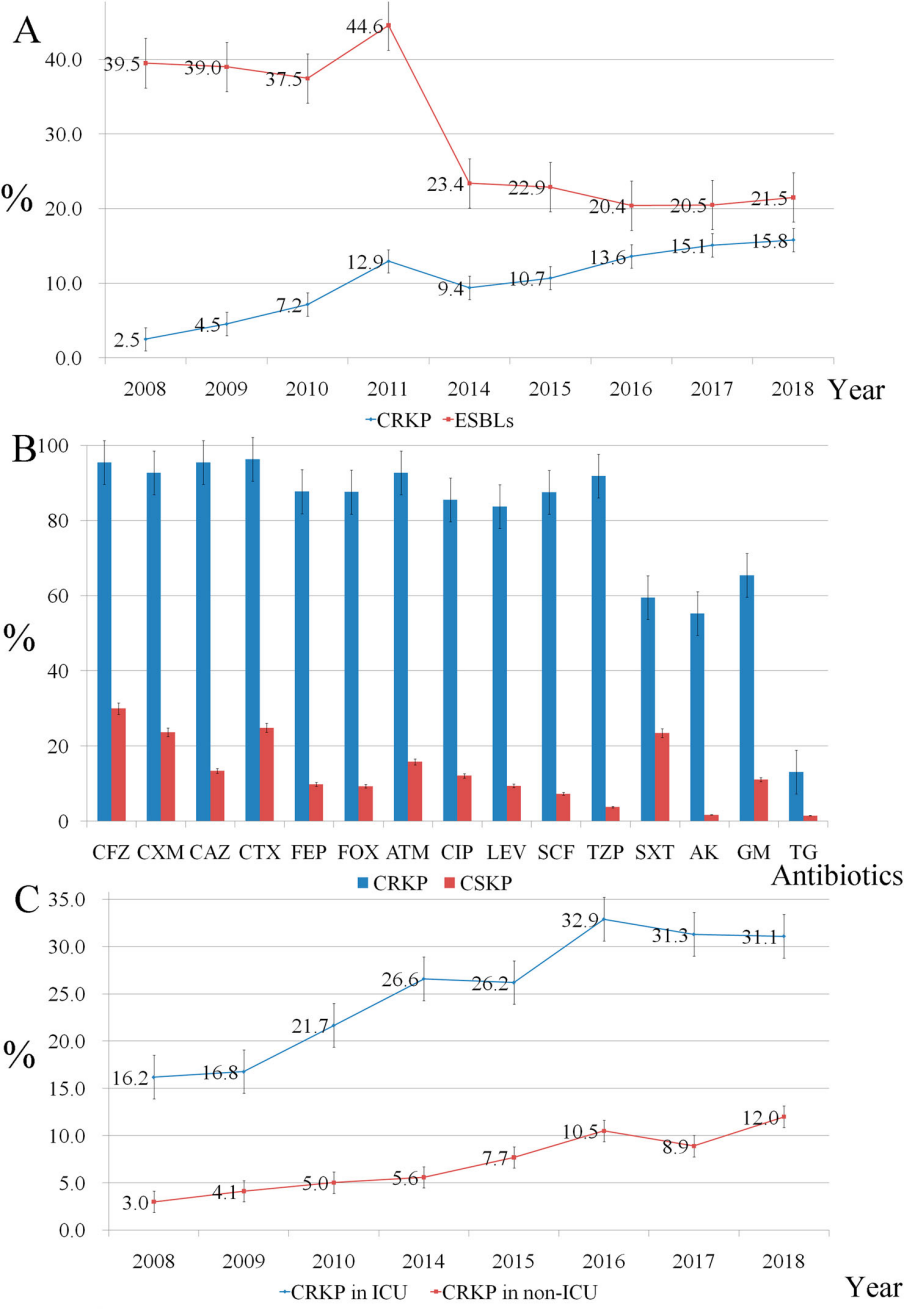
A. Trends in the resistance of *Klebsiella pneumoniae* to extended-spectrum β-lactam (ESBL) and carbapenem from 2008 to 2018. B. Antimicrobial resistance patterns of carbapenem-resistant *Klebsiella pneumoniae* (CRKP) and carbapenem-sensitive *K. pneumoniae* CSKP isolates in 2018. CFZ, cefazolin; CXM, cefuroxime; CAZ, ceftazidime; CTX, cefotaxime; FEP, cefepime; FOX, cefoxitin; ATM, aztreonam; CIP, ciprofloxacin; LEV, levofloxacin; SCF, cefoperazone/sulbactam; TZP, piperacillin/tazobactam; SXT, sulfamethoxazole/trimethoprim; AK, amikacin; GM, gentamicin; TG, tigecycline. C. Comparison of trends in carbapenem resistance in intensive care unit (ICU) and non-ICU wards from 2008–2018.

**Figure 2.**
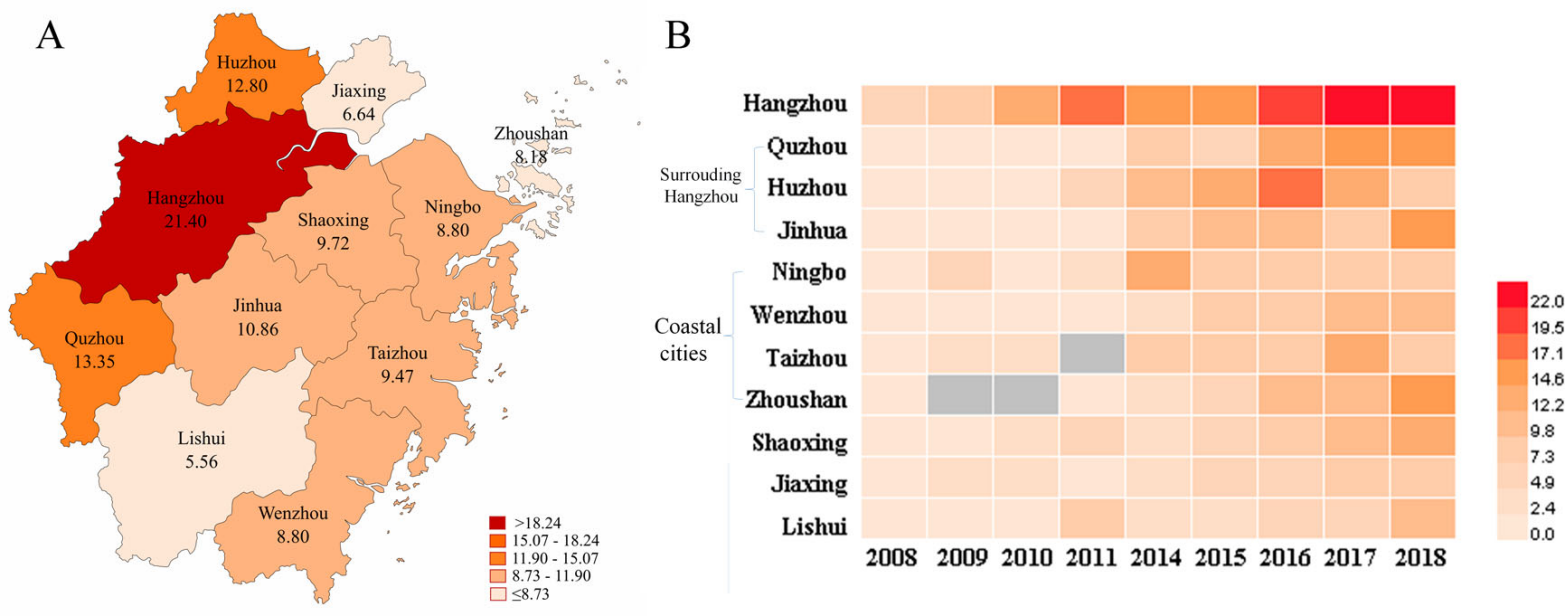
Heatmap of imipenem-resistant *Klebsiella pneumoniae* among the administrative districts of Zhejiang Province. A. Data combined from 2014 to 2018. B. Annual imipenem resistance rates from 2008–2018. The legend shows the corresponding imipenem resistance rates (%). Missing data are marked in grey.

Overall, *K. pneumoniae* showed high rates of susceptibility to lipopeptides and tigecycline (data not shown), and the prevalence of ESBL-producing *K. pneumoniae* decreased from 39.5% in 2008 to 21.5% in 2018 (*P *< 0.001). However, a significant increase prevalence was observed for CRKP, from 2.5% to 15.8% (Figure 1(A)). Cefoperazone/sulbactam resistance levels increased from 9.5% to 21.4%, while piperacillin/tazobactam resistance rates fluctuated between 12.4% and 17.8% throughout the study period. Levels of resistance to cephalosporins, aminoglycosides, and fluoroquinolones slowly decreased between 2008 and 2018 (Table 2), while cefoxitin resistance remained fairly stable (between 17.3% and 21.8%). When we classified the isolates from 2018 into CRKP and CSKP groups, we found significant differences in resistance rates between the two groups for all of the analysed antibiotics (*P *< 0.001) except lipopeptides. CRKP isolates exhibited significantly lower levels of susceptibility to all antibiotics except lipopeptides compared with the CSKP isolates (Figure 1(B)). Moreover, the CRKP isolation rate from ICU wards was significantly higher than that from non-ICU wards. The prevalence of CRKP reached a high of 31.1% in 2018 (Figure 1(C)). Comparison of CRKP isolation rates among the 11 cities in Zhejiang Province showed that CRKP was significantly more prevalent in Hangzhou compared with the other cities (Figure 2(A)). CRKP was not detected in some cities in the first half of the surveillance period (Figure 2(B)).

**Table 2.** Rates (%) of *Klebsiella pneumoniae* resistance to antimicrobial agents from 2008 to 2018.

Antibiotica	Year								
	2008	2009	2010	2011	2014	2015	2016	2017	2018
IMP	2.5	4.5	7.2	12.9	9.4	10.7	13.6	15.1	15.8
MEM	2.9	4.0	8.0	11.5	8.2	11.6	15.0	17.4	20.9
SCF	9.5	10.2	11.3	12.0	-	16.8	17.5	18.7	21.4
TZP	17.4	16.5	17.5	17.8	12.5	12.4	13.8	15.5	17.2
CFZ	50.2	48.8	51.2	54.0	39.7	37.1	40.2	42.3	42.3
CXM	48.3	45.9	48.4	44.1	30.8	32.6	28.4	31.6	32.8
CAZ	41.5	39.2	41.6	33.2	21.6	22.0	24.7	25.2	27.3
CTX	42.8	42.9	44.1	44.5	31.1	39.8	34.1	35.5	37.1
FEP	40.1	38.8	40.4	27.8	17.7	17.8	18.8	20.6	22.0
ATM	44.3	37.6	41.5	36.1	24.1	24.7	24.7	26.2	28.1
FOX	20.4	18.3	19.0	19.4	17.3	18.3	19.9	20.9	21.8
CIP	31.8	27.1	30.7	25.7	19.6	19.0	20.8	22.3	24.0
LEV	24.0	22.6	27.3	20.7	16.0	16.2	18.4	19.1	20.9
AK	12.2	13.0	12.9	10.9	5.7	6.6	8.3	9.4	9.9
GM	29.0	29.6	29.6	26.6	17.4	17.4	18.3	19.1	19.7
SXT	36.4	36.1	34.8	33.6	24.6	25.4	27.0	28.8	29.2

^a^IMP, imipenem; MEM, meropenem; SCF, cefoperazone/sulbactam; TZP, piperacillin/tazobactam; CFZ, cefazolin; CXM, cefuroxime; CAZ, ceftazidime; CTX, cefotaxime; FEP, cefepime; ATM, aztreonam; FOX, cefoxitin; CIP, ciprofloxacin; LEV, levofloxacin; AK, amikacin; GM, gentamicin; SXT, sulfamethoxazole/trimethoprim.

### Risk factors associated with CRKP

The correlation between CRKP and seven risk factors was examined by OR analysis (Table 3 and Supplemental Table S1). The isolation time had a significant effect on the prevalence of CRKP (eg., OR = 7.473 (95% CI, 6.870, 8.129) for 2018 versus 2008). In particular, *K. pneumoniae* isolated during the first and second quarters were more likely to be resistant to imipenem. With respect to location, we determined that all cities had a higher proportion of CRKP isolates compared with Lishui, especially Hangzhou (the capital city of Zhejiang). Further, the hospital level IIIA had a higher proportion of CRKP compared with all other levels. CRKP was also more prevalent among *K. pneumoniae* isolates from ICU wards and inpatients compared with those from non-ICU wards and outpatients, respectively. No significant differences were observed between patient age groups 0–2 years and 3–9 years, while for other age groups, the OR increased along with age, with the highest proportion of resistant isolates observed among those ≥60 years. In addition, isolates from blood and urine were more likely to be resistant to carbapenem than those from sputum cultures (Table 3).

**Table 3.** Analysis of risk factors associated with imipenem-resistant Klebsiella pneumoniae*.

	OR (95% CI)^a^	*p*-value^b^		OR (95% CI)	*p*-value
District			Age (years)		
Lishui	1	NA^c^	0–2	1	NA
Hangzhou^d^	4.65 (4.15, 5.22)	**<0**.**001**	3–9	1.12 (0.85, 1.49)	0.422
Huzhou	2.52 (2.20, 2.90)	**<0**.**001**	10–19	3.30 (2.65, 4.10)	**<0**.**001**
Ningbo	1.65 (1.46, 1.87)	**<0**.**001**	20–39	5.09 (4.34, 5.98)	**<0**.**001**
Taizhou	1.78 (1.57, 2.03)	**<0**.**001**	40–59	5.79 (4.97, 6.75)	**<0**.**001**
Zhoushan	1.50 (1.27, 1.77)	**<0**.**001**	>=60	7.02 (6.04, 8.16)	**<0**.**001**
Wenzhou	1.64 (1.44, 1.86)	**<0**.**001**	**Quarter**		** **
Quzhou	2.61 (2.28, 2.99)	**<0**.**001**	Jul–Sep	1	NA
Jinhua	2.08 (1.84, 2.35)	**<0**.**001**	Jan–Mar	1.97 (1.88, 2.07)	**<0**.**001**
Shaoxing	1.84 (1.62, 2.09)	**<0**.**001**	Apr–Jun	1.62 (1.54, 1.70)	**<0**.**001**
Jiaxing	1.21 (1.07, 1.38)	**0**.**003**	Oct–Dec	1.15 (1.09, 1.21)	**<0**.**001**
**Specimen type**			**Hospital level**		
Sputum	1	NA	IIB^e^	1	NA
Blood	1.20 (1.13, 1.26)	**<0**.**001**	IIA	1.48 (0.87, 2.51)	0.165
Urine	1.39 (1.34, 1.45)	**<0**.**001**	IIIB	1.14 (0.67, 1.94)	0.785
**Type of patient**			IIIA	1.83 (1.08, 3.11)	**0**.**027**
outpaitent	1	NA	**Ward**		
inpatient	4.13 (3.71, 4.60)	**<0.001**	non-ICU	1	NA
			ICU	4.40 (4.24, 4.56)	**<0**.**001**

^a^CI = confidence interval; OR = odds ratio.

^b^NA = not available.

^c^*P*-values < 0.05 are shown in boldface.

^d^Shading indicates that the OR value for this risk factor is >1 for both overall and annual data.

^e^IIIA = grade A class three hospital; IIIB = grade B class three hospital; IIA = grade A class two hospital; IIB = grade B class two hospital. Hospital classifications are determined by the Administrative Agency of the Chinese Government based on bed numbers and comprehensive evaluation scores. Comprehensive evaluation covers number of departments, staffing, management, technical level, work quality, and support facilities. Classifications are as follows: Grade A class three hospital: >500 beds, comprehensive evaluation score >900 points; Grade B class three hospital: >500 beds, comprehensive evaluation score between 750 and 899 points; Grade A class two hospital: 100–499 beds, comprehensive evaluation score >900 points; Grade B class two hospital: 100–499 beds, comprehensive evaluation score between 750 and 899 points.

*Data were collected from 2014 to 2018. Isolates from patients with missing values for specific variables were not included in the analysis.

### Molecular epidemiology of CRKP

**Figure 3.**
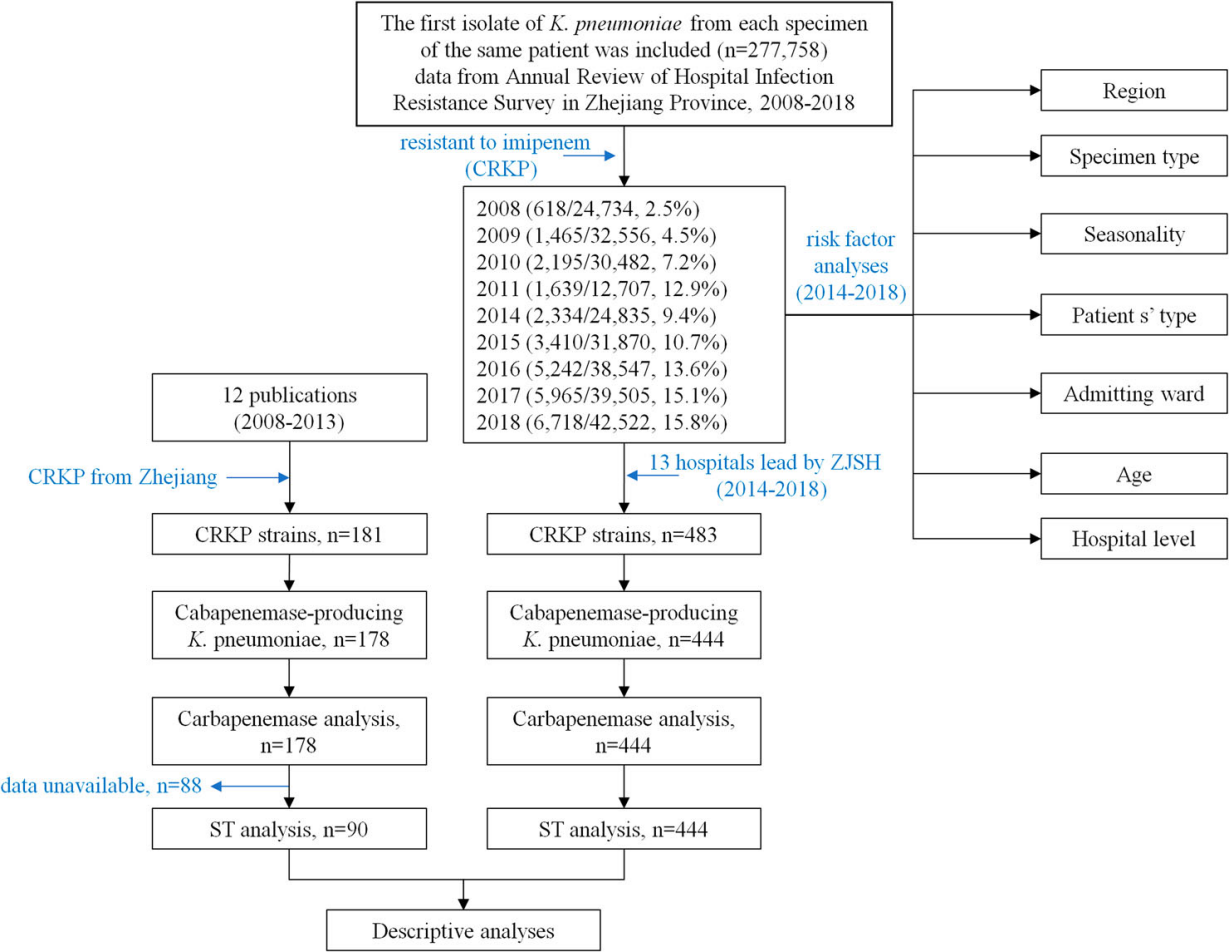
Flow chart diagram of samples in this study. All the strains were collected from clinical samples in Zhejiang. Isolates with missing data were excluded for risk factor analysis. ZJSH, Second Affiliated Hospital of Zhejiang University.

**Figure 4.**
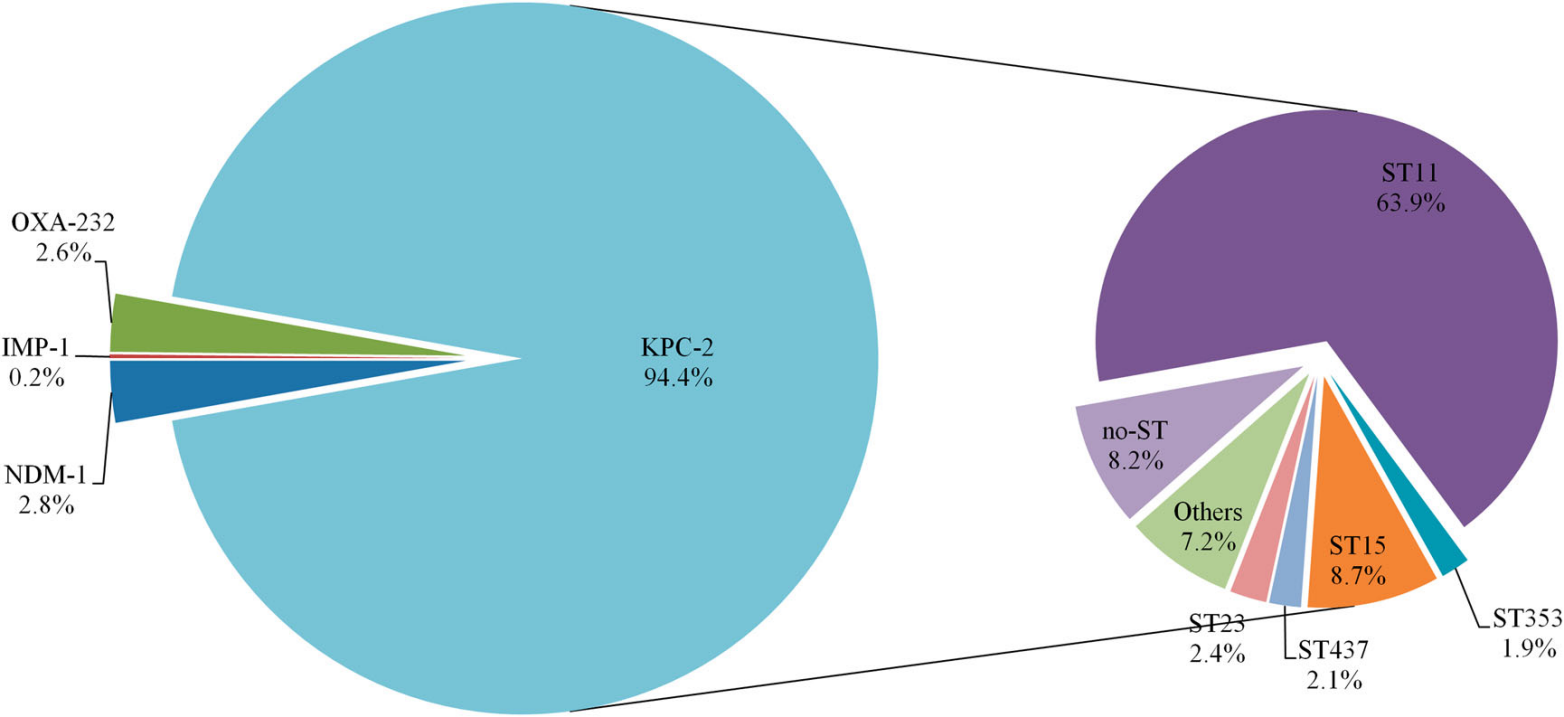
Distribution of carbapenemase genes and sequence types (STs).

We found only 12 studies published between 1 January 2008 and 31 December 2013 that stated specific numbers of CRKP*.* Four studies only involved Zhejiang Province, while five studies only involved other Chinese provinces. Three studies involved both Zhejiang Province and other provinces in China. Three studies described the predominant STs of the CRKP isolates, and all 12 studies described resistance determinants (Figure 3). None of the CRKP isolates from Zhejiang Province collected from 2008 to 2013 contained *bla*_NDM_ variants. Molecular epidemiological analysis revealed that the predominant CRKP ST was ST11, followed by ST15. *bla*_KPC-2_ was responsible for the observed resistance in most of the CRKP isolates across the 11-year surveillance period (Figure 4). Clonal dissemination of NDM-1-producing CRKP and OXA-232-producing CRKP was discovered in 2014–2015 and 2018, respectively. In addition, a ST11 *K. pneumoniae* isolate recovered in 2018 was shown to harbour both *bla*_NDM-1_ and *bla*_KPC-2_ (Supplemental Table S2).

## Discussion

Our study on clinical CRKP in Zhejiang province revealed that the carbapenem resistance has significantly increased over the last several years, and absolute numbers of *K. pneumoniae* isolates from continuously participating hospitals almost doubled between 2008 (*n* = 24,734) and 2018 (*n* = 42,522). Nevertheless, the increase of CRKP was accompanied with the decrease of ESBLs-producing *K. pneumoniae*, not only in Zhejiang, but also in China (http://www.chinets.com/). However, this decreasing trend of ESBLs-producing *K. pneumoniae* might be unreal, as some strains may harbor both carbapenemase- and ESBLs-encoding genes, the phenotype of ESBLs were normally ignored in such situation.

We identified that the *K. pneumoniae* from inpatients and ICU wards were more likely to be imipenem-resistant than isolates from outpatients and non-ICU wards, respectively, which is in accordance with previous studies [7,13] and indicates that ICU admission is an important risk factor associated with CRKP. The CRKP infection is confirmed to be an imminent threat to ICU patients because of the ability of the pathogen to contaminate environmental surfaces in hospital wards, increasing the likelihood of transmission among patients, ICU staff, and the environment [14]. As shown previously [15], our data also demonstrated that patient age is also an independent risk factor for CRKP, which may result from the decreased immune function of elderly patients. It should be note that our isolation rate of imipenem-resistant *K. pneumoniae* of patient age 0–9 was approximately 2.5% from 2014 to 2018, while the CHINET surveillance data showed a 12.6% imipenem-resistant rate of children from 2012 to 2014 [16]. This may be due to that the CHINET system only involved the tertiary hospitals, while our surveillance programme includes both tertiary and secondary hospitals, further manifesting that hospital level is associated with the prevalence of CRKP.

The observation of more prevalent of CRKP in Hangzhou than other cities, and in level 3A hospitals than non-3A hospitals may be attribute to the larger number of critical or immunocompromised patients treated in Hangzhou and at level 3A hospitals. In Zhejiang, the tertiary hospitals are mainly concentrated in Hangzhou, and these are usually level 3A hospitals. As such, many critically ill patients from surrounding cities are transferred to Hangzhou for further treatment. Additionally, we found that CRKP were more prevalent in the cities surrounding Hangzhou such as Huzhou, Quzhou, and Jinhua, which indicates possible transmission of antimicrobial-resistant strains from Hangzhou. In addition, our results revealed that the prevalence of CRKP in coastal cities was higher than that in mountainous areas. It is unknown how the coastal cities associated with the high CRKP occurrence, but the finding could potentially inform clinical recommendations.

Among the different specimen types examined in this study, our results showed that *K. pneumoniae* isolates from urine were much more likely to be carbapenem-resistant than those isolated from sputum or blood. Although studies examining risk factors associated with CRKP carriage are limited, one study of ESBL-producing *K. pneumoniae* demonstrated that the isolation rate of ESBL-positive isolates from urine samples was significantly higher than those from blood and sputum [17].

Previous studies have also shown that the incidence of *K. pneumoniae* infection is associated with the season [18], with isolation rates generally higher in summer than in winter [19]. However, we observed a higher prevalence of CRKP in winter than in summer (data not shown). Although the specific association between the time of year and the incidence of CRKP infection remains unclear, understanding this link could potentially inform clinical recommendations in the future.

Treatment options for CRKP infections are very limited, cephalosporins, β-lactamase inhibitor combinations, fluoroquinolones, and aminoglycosides were not suitable treatment options for most isolates owing to high levels of resistance (Figure 1(B)). Although most of the CRKP isolates were susceptible to polymyxins and tigecycline, surveillance from 2018 reported a tigecycline resistance rate among CRKP isolates of 13.1%, while the efficacy of polymyxins for treating CRKP infections has recently been questioned following clinical monotherapy trials [20]. Polymyxins should generally be used in combination with other antibiotics in the clinical therapy. However, novel FDA-approved antibiotics such as ceftolozane-tazobactam or ceftazidime-avibactam may be alternative options. These drugs have been verified to have good activity against CRKP isolates, especially KPC-producing CRKP. However, they are not as effective against NDM-producing CRKP strains [21]. In China, ceftolozane-tazobactam has not yet been approved, and ceftazidime-avibactam was only approved on 8 September 2019. Unfortunately though, ceftazidime-avibactam-resistant CRKP has already been reported in various countries [22,23]. Therefore, to prevent the development of novel ceftazidime-avibactam-resistant strains in China, identification of the resistance mechanisms should be a prerequisite to developing rational antibiotic regimens.

The KPC-type enzymes are the most prevalent carbapenemases among our tested CRKP isolates. Interestingly, Hangzhou was the first city in China from which a KPC-producing CRKP isolate was reported [9], although KPC-producing CRKP have subsequently been detected in surrounding cities and provinces [11]. This may partially explain the higher prevalence of CRKP in Hangzhou and the surrounding cities in the current study. Although there are reports of other carbapenemase-producing CRKP strains as well as the clonal transmission of these carbapenemases [8,24], KPC-type enzymes have always been the predominant carbapenemases in CRKP, particularly among ST11 KPC-producing strains. We recently reported a fatal outbreak of ST11 carbapenem-resistant hypervirulent *K. pneumoniae* [25], and suggest that future surveillance studies pay special attention to ST11 KPC-producing strains.

Together, our findings provide valuable information for the development and implementation of infection control practices. Most of the surveillance studies conducted to date in China only covered tertiary hospitals and lack molecular data. In our study, resistance was observed in a wide range of hospitals, indicating the importance of performing regional antibiotic resistance surveillance. However, our study had several limitations. First, for the classification analysis based on age, patient type, or isolation time, isolates without the corresponding field were excluded, which may have introduced bias to the resistance rate calculations. Second, the level of hospitals that participated in the surveillance was uneven. Some hospitals were inevitably biased towards having a statistically significant antibiotic resistance rate because of the small number of isolates from that hospital. Third, the molecular mechanisms of carbapenem resistance among the CRKP isolates were not available from the surveillance data. Therefore, we used the reported molecular data from the studies published between 2008 and 2013 along with further analysis of the isolates collected from the surveillance hospitals between 2014 and 2018 to fill in the blanks in the molecular epidemiology analysis.

In conclusion, our study reveals increasing trend and molecular features of CRKP isolates in Zhejiang Province, China. Five demographic factors associated with higher CRKP infections provide important perspectives into CRKP and highlight the urgent need for medical institutions to strengthen their surveillance of CRKP in China.

## Materials and methods

### Bacterial strains and species identification

Data from patients were obtained from the Annual Review of Hospital Infection Resistance Survey in Zhejiang Province, 2008–2018 (data from 2012 and 2013 were unavailable, Figure 3). All hospitals involved in the study are secondary or tertiary hospitals and are accredited to perform pathogen identification and antimicrobial susceptibility testing. Participation by the laboratories was voluntary and changed over time. The hospitals are distributed across all 11 cities in Zhejiang Province, including Hangzhou, Huzhou, Jiaxing, Shaoxing, Ningbo, Taizhou, Jinhua, Quzhou, Lishui, Wenzhou, and Zhoushan. Isolates were identified by matrix-assisted laser desorption/ionization time of flight mass spectrometry (MALDI-TOF MS) (Bruker Daltonik GmbH, Bremen, Germany), the Vitek 2 Compact system (bioMérieux, Durham, NC, USA), or the Phoenix100 system (Becton Dickinson, Sparks, MD, USA) as per the manufacturers’ instructions.

### Antimicrobial susceptibility testing

Antimicrobial susceptibility testing of the *K. pneumoniae* isolates to common clinically used antibiotics was performed via the Kirby-Bauer method according to a Clinical and Laboratory Standards Institute (CLSI) unified protocol, or by the broth microdilution method using automated systems (Vitek 2 Compact system or Phoenix100 system). To allow comparison of the results of identification and susceptibility testing among hospitals, the same reference strain and standard operating procedures were used for each method, as suggested by the National Health Commission of the People’s Republic of China. The same specimen type was analysed from each patient, and only the first isolate from each specimen (one per patient) was selected. Susceptibility results were interpreted according to the annually CLSI-M100 documents (https://www.clsi.org/). *K. pneumoniae* ATCC 13883 was used as the quality control strain.

### Classification of the variates and statistical analysis

Statistical analysis of the variates was based on the data from 2014 to 2018. To eliminate year as a factor, the variates were calculated both annually and across all years. Specifically, the geographic regions were grouped through each city. Age was classified into a categorical variable for the data analysis (≤2, 3–9, 10–19, 20–39, 40–59, and ≥60 years). The influence of specimen type, including blood, sputum, and urine, was also analysed. Outpatients and inpatients were analysed separately, and the inpatients were further divided into ICU and non-ICU groups. To assess the seasonality of CRKP, a categorical variable was created based on four quarters: January–March, April–June, July–September, and October–December. For all data, unconditional logistic regression models were used to estimate odds ratios (ORs) and 95% confidence intervals (CIs) for univariable analysis of risk factors associated with imipenem-resistant *K. pneumoniae* isolates. Pearson’s chi-square (*χ*2) test or Fisher’s exact test were used to compare the resistance levels of different groups, with *P* < 0.05 considered statistically significantly. All statistical analyses were two-tailed and were performed using Statistical Package for the Social Sciences version 23.0 (SPSS Inc., Chicago, IL, USA). In this study, meropenem and ertapenem resistance were less common than imipenem resistance. Therefore, imipenem resistance was used as an indicator of carbapenem resistance. Meanwhile, imipenem-resistant isolates were defined as CRKP in the statistical analysis.

### Carbapenemase-encoding genes and sequence type analysis

Carbapenemase gene and sequence type analyses of all isolates recovered from 2014 to 2018 were performed in our laboratory, with isolates obtained from 13 hospitals in Zhejiang Province. All isolates were identified using a MALDI-TOF MS (Bruker Daltonik GmbH, Bremen, Germany). Genomic DNA was extracted from the isolates using a PureLink Genomic DNA Mini Kit (Invitrogen, Carlsbad, CA, USA) according to the manufacturer’s instructions, and then sequenced using the Illumina HiSeq X10 platform with a 150-bp paired-end strategy. Raw reads were trimmed and assembled to contigs using SPAdes version 3.11.1 [26]. Acquired carbapenem resistance genes and multilocus STs were determined using ResFinder 2.1 [27] and Kleborate [28], respectively.

CRKP isolates recovered from 2008 to 2013 in Zhejiang Province were unavailable. Thus, carbapenemase gene and ST data corresponding to this time period were acquired from studies published during this time using isolates from Zhejiang Province. We searched PubMed with no language restrictions for reports that were published from 1 January 2008 to 31 December 2013 using the terms “China and carbapenem-resistant *Klebsiella pneumoniae*,” “China and carbapenem-resistant *Klebsiella pneumoniae* and ST11,” “Zhejiang and carbapenem-resistant *Klebsiella pneumoniae*,” “Zhejiang and carbapenem-resistant *Klebsiella pneumoniae* and ST11,”“China and carbapenem-resistant *Klebsiella pneumoniae* and plasmid,” and “China and ST11 and carbapenem-resistant *Klebsiella pneumoniae* and plasmid” (Supplementary Figure S1).

## Ethics

This study is conducted on already available data from Zhejiang AMR Surveillance Coordination Group (ZASC), and Prof. Rong Zhang also participated in this group. Ethical approval was approved by the Ethics Committee of The Second Affiliated Hospital of Zhejiang University, School of Medicine (Number: 2020-319).

## Data Availability

Supplementary information accompanies the manuscript on the Emerging Microbes & Infections website http://www.nature.com/emi.
